# The Public Health: Interviews with Local Authorities—Middlesex

**Published:** 1923-04

**Authors:** 


### THE PUBLIC HEALTH.

### INTERVIEWS WITH LOCAL AUTHORITIES.

### VIII.—THE COUNTY OF MIDDLESEX.

(By our Special Commissioner.)

Big Ben with one face looks across Parliament Square to the Guildhall which is the headquarters of the County Council of Middlesex. With another face he looks across the river to the home of the London County Council. And as one reflects on this there at once springs to the mind a question which is of the first importance to the people of Middlesex—Is the County of Middlesex a separate community with its own separate problems, its own interests, its own corporate spirit ? Or is it part of that vast, ever-growing monster body which we call London ? It is a question to which one to whom Big Ben is no stranger—Lord Ullswater, as Chairman of the Royal Commission on London Government—will very shortly propound an answer.

### What is Community of Interest ?

We have been favoured with an interesting review of some of the aspects of health administration in Middlesex by Mr. Todd, Chairman of the Health Sub-Committee, who is Vice-Chairman of the County Council, by Dr. Young, the County Medical Officer, and his deputy, Dr. Tate. Underlying all their activities and their zeal for their public work is the feeling—not perhaps expressed in so many words—that the County of Middlesex is a very real corporate entity with its own life and interests, and not at all inclined to become a mere appanage of London. London may claim community of interest, but there is too much reason for thinking that, at any rate where public health problems are concerned, community of interest, when the population is vast, means lack of interest. It may be true that the principal spokesman of the London County Council before the Royal Commission—Mr. Norman—did not lay great stress on the question of public health administration. But are not all problems of local government sooner or later health problems ?

### A Wonderful Growth.

The administrative County of Middlesex has some reason for keeping a watchful eye on London. In the year 1888, at one fell swoop, the newly-formed County of London took from Middlesex 31,484 acres, carrying a population of 2,687,271. With some minor adjustments the area then left has remained the same down to the present time. But the population soon got into its stride as follows : In 1891, it was 543,223 ; in 1901, 792,476 ; in 1911, it had grown to 1,126,694 ; and in 1921, to 1,253,164. In what sense this increase has come about because of London, how far it is due to the displacement of slum areas and to other improvements in the inner ring of London, or how far to the development of industries and growth of factories in the County, it is unnecessary here to examine. It is sufficient to say that the Middlesex man feels that his own County administration is looking after his affairs better than would be the case if it were merged in a Greater London.

### Importance of Co-operation.

What is probably covered by the phrase " community of interest " may be—and, it is claimed, is—secured by co-operation. In the treatment of tuberculosis, for example, a man living in Middlesex at the time of his application for treatment is dealt with on the merits of his case, without the probing of the vexatious question, often almost incapable of settlement—whether he belongs to London or Surrey or Essex. There are smoothly-working arrangements with neighbouring authorities by which proper financial adjustments are made, and there is an interchange in suitable cases of past histories. In regard to venereal disease, there are joint arrangements in force involving, *inter alia*, the use of London hospitals. With regard to infectious diseases generally, there is effective and rapid exchange of information, and co-operation with the neighbouring authorities.

### A Model Tuberculosis Scheme.

The Health Committee claim, not challengingly, but with confidence, in regard to their scheme for the treatment of tuberculosis, that it is a model scheme. This applies, in particular, to their dispensary system, which has recently been the subject of a very full and valuable report by Dr. Young. The dispensary is, as it was always contemplated that it should properly be, an organisation and not a building. In this organisation in Middlesex there are engaged six whole-time tuberculosis officers and the necessary ancillary staff. A special feature of the work at the various branch dispensaries is that there is no doling out of drugs to encourage patients to attend. No dispensing of drugs is done. The out-patient room atmosphere is avoided, because—a really excellent feature of the administration—the patients are seen for the most part by appointment. Nearly all the doctors practising in the county consult the tuberculosis officers, or readily refer their patients to the dispensaries for assistance in diagnosis and treatment. The patients willingly continue in attendance. There is systematic home-visiting by nurses trained to give advice as to hygienic modes of living and home conditions. Prevention plays no mean part in the working of the dispensary ; this consists especially in the examination of contacts and suspects. It is satisfactory to learn in connection with the bacteriological examinations of sputum generally that in quite a high proportion of the cases examined negative results are given. In many of these cases the expense and anxiety to the patient of a stay in an institution for the purposes of observation might have been involved, but for an effective dispensary organisation. Middlesex has made generous provision in the past for the treatment of selected cases in beds reserved in sanatoria and other institutions up and down the country. They now have a fine sanatorium of their own at Harefield, containing a children's block,

64 beds with classrooms, dining, recreation and day rooms, a nursing block, 46 beds, for the more serious cases, male and female, a women's block, 106 beds, and a men's block, 106 beds; a total of 322 beds.

### Colony Treatment.

The Committee's experience of farm colony treatment which has been undertaken for ex-Service men has so far been disappointing. "I do not know," says one of the Council's tuberculosis officers, "of any man, of the type concerned, who would consent to the permanent uprooting entailed by settlement in a farm colony." He considers that the colony has no adequate advantage over a sanatorium where there is a proper graduated labour system.

### Maternity and Child Welfare.

The Council have made provision for midwifery services in areas insufficiently supplied by appointing three whole-time midwives and by subsidising one in another area. The provision of maternity hospital accommodation for women has also been considered, and an adequate scheme was drawn up to provide such institutions in conjunction with some of the adjoining District Councils. The carrying out of these schemes has been postponed owing to the need for economy. The provision of dental treatment for nursing and expectant mothers and children under five years of age was started, but in the middle of 1921 this work also was suspended on grounds of economy. The cold hand of economy has also arrested the development for the time being of such services as hospital treatment for cases of complicated confinement, the provision of day nurseries, of home helps, of convalescent homes for children under five, &c., but it is hoped that these services, which are all provided for in the scheme adopted by the Council, will not be much longer delayed. Meanwhile the work proceeds apace at the various centres with which the county is covered. There were 53,788 attendances in 1921. Stress is laid on the educational character of these centres, and on the fact that their essential function is to supervise the healthy child rather than to treat the sick. Dr. Young is the County School Medical Officer, and there is in general a unification of the respective medical and nursing services.

### Too Many Maps of Middlesex.

While it is true that the relations of the County Health Committee with all the other different authorities in the county having health functions are excellent, one cannot fail to be impressed with the fact that here as elsewhere there are too many health authorities—too many maps of Middlesex. We invited Mr. Todd, out of his wide experience and interest in problems of local government, to express his views on the need for one co-ordinating authority for all health services in the county. He did not think that this could seriously be called into question, but he very sagely observed that a condition precedent must be a uniform basis of valuation, and that the bringing of county health services under one general control would have to be accompanied by real, effective delegation.

